# The Impact of the COVID-19 Pandemic on STI and HIV Services in the Netherlands According to Health Care Professionals

**DOI:** 10.3390/ijerph21060678

**Published:** 2024-05-25

**Authors:** Annemarie Reilingh, Jenneke Van Ditzhuijzen, Thijs Albers, Hanna Bos, John De Wit

**Affiliations:** 1 Department of Obstetrics and Gynecology, Amsterdam University Medical Center, Meibergdreef 9, 1115 AZ Amsterdam, The Netherlands; j.m.vanditzhuijzen@uu.nl; 2 Department of Interdisciplinary Social Science, Utrecht University, Heidelberglaan 8, 3584 CS Utrecht, The Netherlands; 3 Soa Aids Netherlands, Condensatorweg 54, 1014 AX Amsterdam, The Netherlands

**Keywords:** COVID-19, STI/HIV care, sexual health, public health service, healthcare professionals, access to healthcare

## Abstract

Surveillance data from the Netherlands show that STI/HIV testing decreased at the start of the COVID-19 pandemic, suggesting barriers to access to STI/HIV care. However, the impact of the pandemic on STI/HIV care may be more complex, and key populations could be differentially affected. The aim of this study was to gain more insight into the impact of COVID-19 on STI/HIV care in the Netherlands from the perspective of STI/HIV care providers. We investigated whether professionals in STI/HIV care experienced changes compared to pre-COVID in access to STI/HIV care for priority populations, demand and provision of STI/HIV care, shifts to online STI/HIV counseling and care, and the quality assurance of STI/HIV care. An online survey was completed by 192 STI/HIV care professionals. Additionally, semi-structured interviews were held with 23 STI/HIV care professionals. According to participants, people in vulnerable circumstances, such as recent migrants and people with low health or digital literacy, may have had difficulties accessing STI/HIV care during the pandemic, especially during lockdowns and at public sexual health services. Hence, these may not have received the care they needed. Participants thought that COVID-19 measures may have compounded existing disparities. Furthermore, participants found that online care provision was not up to standard and were concerned about follow-up care for patients using private online providers of remote tests. It is important to explore how STI/HIV care for people in vulnerable circumstances can be ensured in future public health crises.

## 1. Introduction

The COVID-19 pandemic has posed major challenges to healthcare systems globally, including prevention, diagnosis, and treatment of STIs and HIV in the European region [1,2,3]. The impact of the pandemic on both demand and provision of STI/HIV care and how the pandemic affected healthcare use and STI/HIV transmission is complex and may not be captured by surveillance data alone. Additional research is needed to gain insight into the nature of changes to healthcare that may have occurred during the COVID-19 pandemic.

Several international reviews have assessed the impacts of the COVID-19 pandemic on STI/HIV services [4,5,6]. Changes in sexual behavior and healthcare service provision and use during the COVID-19 pandemic may have had differential effects and complex combined impacts [5]. Changes that were noted in the international literature include altered health-seeking behavior, reduction of face-to-face contact, stricter triage, and clinic staff being redeployed to assist COVID-19 control efforts [4,6]. To mitigate some of these effects, the use of online consultations and the use of remote self-testing has increased and may continue to have a place in post-pandemic STI/HIV care provision. In the Netherlands, it remains unknown to what extent the COVID-19 pandemic has affected STI/HIV care, especially for vulnerable populations.

There are four types of STI/HIV health service providers in The Netherlands. The general practitioner (GP) is the main point of access for STI/HIV prevention, diagnosis, and STI treatment [7]. HIV treatment is provided through specialized HIV clinics [8]. In addition, the Netherlands has a network of publicly funded regional public sexual health services (PSHS) that offer STI/HIV prevention, diagnosis, and treatment for key populations at risk: men who have sex with men (MSM), sex workers, and young people under the age of 25 [9]. Lastly, over the last decade, online private providers have started to offer patient-initiated remote STI/HIV testing at commercial rates. Private providers that meet quality indicators have been identified by Soa Aids Nederland, the center of expertise on STI and HIV in the Netherlands.

In the spring of 2020, sexual health services at PSHS were scaled down due to COVID-19. The PSHS had capacity issues related to staff deployment in COVID-19 control, and efforts were made to reduce face-to-face contact to avoid the spread of the virus. During periods with high restrictions, clients could visit the PSHS only for essential care, limited to consultations for clients who had (severe) STI-related complaints, were in need of treatment, received an STI partner notification, or had experienced sexual violence. Outreach activities for people in vulnerable conditions were largely discontinued, and people were encouraged to avoid unnecessary use of healthcare, including their GP.

More insight is needed into how the COVID-19 pandemic affected STI/HIV care and the specific challenges healthcare professionals may have faced. The objective of this study is to investigate professionals’ experiences during the COVID-19 pandemic regarding access to STI/HIV care for priority populations, demand and availability of STI/HIV care, shifts to online STI/HIV counseling and care and the quality assurance of STI/HIV care. This knowledge is relevant to improving the pandemic-preparedness of STI/HIV services so that delivery of STI/HIV care is guaranteed as much as possible during future public health crises.

## 2. Methods

### 2.1. Study Design

Between 20 October 2021 and 1 January 2022, we conducted an online survey with STI/HIV care professionals from GP practices, PSHS, and HIV clinics via a secure Qualtrics platform. We asked survey participants to rate various aspects of STI/HIV care during four COVID-19 periods in comparison to pre-COVID-19. The study also included semi-structured interviews with STI/HIV care professionals from GP practices, PSHS, and HIV clinics, but also from private testing providers to provide further rich information from a first-person perspective. Interviews were held between October 2021 and January 2022, live or via secure video conferencing platforms (Teams or Zoom), and lasted about 35–45 min. All participants provided full informed consent before any data were collected and were advised that they could stop participation at any time. The research team was supported by an advisory committee consisting of experts in STI/HIV care from PSHS, a GP practice, Soa Aids Nederland, and the Dutch National Institute for Public Health and the Environment (RIVM). The survey questionnaire and interview topic list were developed in collaboration with the advisory committee. The study protocol received approval from the Human Research Ethics Committee of the Faculty of Social and Behavioral Sciences, Utrecht University (file number 21/0268).

### 2.2. Participants and Recruitment

Survey participants were recruited through various professional networks of STI/HIV care providers (i.e., GP-web, the sexual health expert group of the national GP federation, the Dutch association of GP assistants, the sexual health physicians working group of the PSHS, the national network of PSHS managers), the annual Dutch national STI/HIV and sexual health conference, and LinkedIn pages of members of the study’s advisory committee. Participants in the interviews were recruited through professional networks and associations of GPs and nurses specialized in STI/HIV, PSHS, and the study’s advisory committee. Participants in the survey and the interviews were also asked to circulate our call for participants in their networks (i.e., snowballing).

### 2.3. Online Survey

Survey questions were asked for four periods that broadly reflected the evolving COVID-19 restrictions in the Netherlands: the period of the first lockdown (mid-March–early summer 2020), the relatively restriction-free summer of 2020, the period of the second lockdown (fall 2020–spring 2021), and the period of easing restrictions (spring 2021–fall 2021).

Ten questions measured observed changes in STI/HIV care in the participants’ healthcare practice since the start of COVID-19 compared to before. Four questions addressed the estimated number of face-to-face contacts, telephone or online consultations and referrals to private testing providers and perceived access for key populations (youth, men having sex with men (MSM), sex workers), for example, ‘The access to STI/HIV care for sex workers in my practice was…’. Participants were asked to rate each item for each of the four periods, compared to pre-COVID-19, with responses given on a five-point scale from 1 (a lot less) to 5 (a lot more).

Six questions, phrased as statements, assessed participants experienced workload, opportunity for 1-to-1 counseling, peer-to-peer coaching and education, and job satisfaction and were rated on five-point scales ranging from 1 (strongly disagree) to 5 (strongly agree), for example, ‘The demand for STI/HIV care was bigger than we could offer’, ‘STI/HIV care has become more efficient’. Responses to these statements were also obtained for all four time periods.

Participants’ age, the healthcare setting in which they worked, the geographical location of the setting (province), and their specific occupations were also assessed.

### 2.4. In-Depth Interviews

Semi-structured interviews were conducted by the first three authors. We used a semi-structured interview guide covering questions similar to those in the survey, creating the opportunity to probe further and collect richer answers to increase understanding of the survey. Interviews started with questions about pre-pandemic STI/HIV care in participants’ healthcare setting in terms of types of care provided, number and nature of consultations, and number and types of clients. Then, a timeline was shown, and we asked the interviewees to describe what happened in each of the four stages of the COVID-19 pandemic, starting with the first lockdown. After this, participants were asked to describe whether and how, from their perspective, COVID-19 measures may have affected access to STI/HIV services, demand for (and provision of) STI/HIV testing and care, potential shifts in care, and quality of care. Lastly, interviewees were asked to think about the future of STI/HIV care and suggest possible ways to ensure access to STI/HIV care and the quality of care in future public health crises.

### 2.5. Data Analysis

Survey data were analyzed in SPSS Statistics (version 27). Missing values were excluded by variable to retain as much data as possible. Repeated measures analyses of variance (RM ANOVA) were conducted to assess differences in responses across the four periods. Differences between GP practices and PSHS were also tested. Bonferroni correction of tests of between-group differences was used to limit the occurrence of chance effects. Interviews were transcribed verbatim, and transcripts were mostly coded in NVIVO, with some manual coding. Most of the coding was performed by TA; coding of interviews with private providers was performed by JVD. Coding was primarily deductive, based on the research questions. Emerging (sub)topics were coded inductively [10]. We discussed the coding and eventual themes amongst three of the authors (JVD, TA, AR) and with experts in the advisory committee

## 3. Results

### 3.1. Participant Characteristics

**Figure 1:**
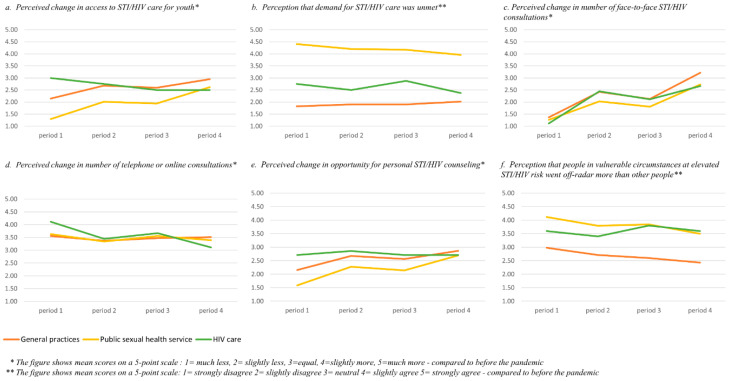
Perceived changes during four periods of the COVID-19 pandemic in GP, PSHS, and HIV care (selection).

In total, 197 STI/HIV care professionals accessed the survey. Those who did not complete any survey questions (n = 5) were excluded, resulting in a study sample of 192 participants. Of these, 77 (40%) worked in GP practices, 93 (48%) in PSHS, 13 (7%) in HIV clinics, and 9 (5%) worked in other STI/HIV care settings. The mean age of the survey participants was 44.4 years (SD = 11.6). We excluded participants working in other STI/HIV care settings, as this was a small group and the variety of professions was large (i.e., pharmacist, social worker). The number of professionals working in HIV clinics was too small to include in the RM ANOVAs, which hence were only performed to assess differences between participants working in GP practices and PSHS. In Table 1, all survey means (and SDs) are presented for these two groups only. Even though the survey results from HIV clinics could not be included in the RM ANOVAs, we considered them to be informative, and they are in line with the data from the in-depth interviews. Therefore, we also included a Figure (Figure 1) in which over time fluctuations in the four COVID-19 periods for all three groups (GP, PSHS, HIV clinics) are displayed. In the following paragraphs, we start with describing the survey findings on the four main research objectives related to STI/HIV care in COVID-19 times (access for priority populations, demand and availability, shifts to online counseling and care, and quality assurance). First, we describe differences between GP and PSHS professionals, followed by differences between the four periods.

Of the 23 healthcare professionals who participated in the in-depth interviews, five worked in GP practices, five worked in PSHS, eight were HIV consultants, and five were managers at private online testing providers. After describing the survey findings, we present the findings from the interviews in a separate paragraph. Quotes from the interviews are provided in Table 2. For clarity and conciseness, we selected findings from the interviews that offered coherence, novelty, and/or enhanced understanding of the survey results.

### 3.2. Access to Care for Priority Populations

Survey data showed that professionals in GP practices and PSHS both thought access to STI/HIV care for youth, MSM, and sex workers was lower than pre-COVID-19 (see Table 1). In GP practices, perceived lower access was significantly less pronounced than in the PSHS for all three priority populations—e.g., youth (F(1,21) = 34.57, *p* < 0.001), MSM (F(1,111) = 10.03, *p* = 0.002) and sex workers (F(1,91) = 19.07, *p* = 0.002). Perceived lower access was most pronounced during the first lockdown compared to other periods and was perceived to have partially recovered in the fourth period when restrictions were relaxed (i.e., the experienced access for youth across time (F(3,363) = 69.51, *p* < 0.001). These differences between time periods were significant, except for the one between the second and third periods. Figure 1 (panel a) also shows that these differences over time were not experienced in the same way by professionals in HIV clinics.

### 3.3. Demand and Availability of STI/HIV Care

In PSHS, professionals more strongly agreed that demand was higher than they could provide, while this was not the case for professionals in GP practices (see Table 1). Figure 1 (panel b) shows that professionals from HIV clinics were in between these two. These differences in the experienced demand and availability of care were significant (F(1,108) = 184.89, *p* < 0.001). Similarly, professionals in PSHS were also more likely to agree than their peers in GP practices that in their setting, fewer people received the care they needed (F(1,103) = 33.06, *p* < 0.001). Only the latter outcome showed significant over time changes: participants felt that especially in the first lockdown, fewer people received the STI/HIV care they needed, but this increased over time (F(3,309) = 12.40, *p* < 0,001).

### 3.4. Shifts to Online Counseling and Care

Professionals, overall, experienced that the number of face-to-face consultations during the COVID-19 pandemic was lower than before (see Table 1). This effect was significantly stronger for professionals from the PSHS compared to those from GP practices (F(1,131) = 10.91, *p* < 0.001. Professionals said the number of face-to-face consultations was the lowest during the first lockdown. We found significant differences between four time periods (F(3,393) = 145.65, *p* < 0.001). Figure 1 (panel c) also shows a similar trend among professionals from HIV clinics.

Professionals experienced a small increase in online consultations during the COVID-19 pandemic compared to before. However, we found no significant differences between the experiences in different sectors (F(1,118) = 0.01, *p* = 0.92, nor between time periods (F(3,354) = 2.15, *p* = 0.09). Professionals in PSHS reported that during COVID-19, they referred clients to private online providers of self-tests more often than before, while those in GP practices did not (F(1,96) = 55.77, *p* < 0.001). Perceived referrals significantly differed between time periods (F(3,288) = 3.11, *p* = 0.03), yet Bonferroni corrected comparisons showed that none of the time periods differed significantly from any other time period.

### 3.5. Quality Assurance of STI/HIV Care

Survey findings show that professionals disagreed with the statement that the quality of care had been less than before the COVID-19 pandemic (see Table 1). There was no difference between professionals from PSHS and GP practices in the perceived impact of the COVID-19 pandemic on the quality of care (F(1,99) = 2.28, *p* = 0.13). We did see more pronounced, significant declines in agreement with this statement across time periods, except between the second and third periods (F(3,297) = 17.08, *p* < 0.001). Professionals said they experienced that they inadequately reached people in vulnerable circumstances compared to pre-COVID. Professionals in PSHS were significantly more likely than those in GP practices to experience that people from key populations in additional vulnerable circumstances were inadequately reached (F(1,91) = 33.15, *p* < 0.001). Across groups, this was experienced more during the first lockdown compared to other periods (F(3,273) = 22.70, *p* < 0.001).

### 3.6. Results from the In-Depth Interviews

With regard to access to care, the in-depth interviews showed that PSHS professionals attributed the experienced lowered access to STI/HIV care mainly to stricter triage rules and the temporary interruption of outreach activities. PSHS professionals were especially concerned about access and care for individuals in key populations with additional vulnerabilities, such as lower health or digital literacy, limited understanding of the Dutch healthcare system, and language barriers (see Table 2). Furthermore, interviewees from HIV clinics confirmed that access to HIV care was not lower in specialized HIV care, potentially because this was not scaled down. However, they did mention that they may have been harder to find by new migrants, and also that their clients who would normally go to the PSHS, now had nowhere to go (Table 2).

With regard to demand and availability of care, professionals in GP practices attributed the lower demand during the first lockdown to lower levels of sexual activity among their clients and to government communication to ‘not unnecessarily burden care’ (see quote in Table 2). In the fourth period, professionals thought that the demand had changed back to almost normal again, but GP practices were overburdened due to delayed care. Professionals in PSHS felt they could not meet demands due to restrictions and staff deployment to the COVID-19 response. While during the first stages of the COVID-19 pandemic, PSHS professionals said they were unable to meet demand, those from GP practices and private online providers said they may have been able to provide more care. Professionals mentioned there was a desire for centralized coordination and communication about which (key) populations are seen by which professionals.

Shifts to online counseling and care were experienced in different ways and to various degrees by different groups of providers. In the interviews, most private online testing providers stated they experienced increased demand for testing since the start of the COVID-19 pandemic and attributed this to lowered access to public healthcare. Professionals from all other services reported some shifts to online counseling but mostly mentioned challenges with the available ICT infrastructure, including problems with internet connectivity, outdated ICT systems (for both professionals and users), problems using video conferencing software, and difficulties accessing electronic patient files (see Table 2). Privacy was also a concern for remote consultations by phone or video call when clients shared living spaces with others. In addition, professionals found the shift to phone or video call consultations problematic because of the limited potential to take non-verbal cues into account. They also found that specific, more sensitive issues (i.e., mental health, addiction, sexual violence, contraception) are more appropriately addressed in face-to-face consultations than by phone or video call.

Focusing on the quality of care, professionals expressed concerns about the accumulation of complex medical problems that may be caused by delays in providing STI/HIV care (see Table 2). According to professionals, barriers to care may have become higher for people who are harder to reach, such as new migrants or sex workers. A further concern was that professionals experienced large variations in the quality of follow-up care offered by private online providers after a positive STI or HIV test.

## 4. Discussion

Findings from the survey and interviews highlight that healthcare professionals perceived lower access to STI/HIV care for key populations during the COVID-19 pandemic. This was especially the case in PSHS. Professionals assumed this to be related to stricter triage and the discontinuation of outreach activities, as well as online consultations. Especially during the first lockdown, demand for care was higher than the availability of care in the PSHS, but this was not the case for professionals from GP practices. In line with this, STI/HIV surveillance data from the Netherlands show a decrease in the number of consultations and tests provided, especially during the first lockdown, but a stable number of recorded positive STI tests, both in GP practices and PSHS [11]. This suggests that STI/HIV care providers have been successful in prioritizing key populations for STI/HIV, such as MSM. However, surveillance data do not provide information on the number of diagnoses potentially missed and the impact of missed diagnoses on the transmission of STIs and HIV [12,13].

We also found that providers working in HIV clinics experienced that access to HIV care in their settings remained quite constant. Other international studies found that access to anti-retroviral drugs, (periodic) HIV testing, HIV prophylaxis, and counseling services were affected during the COVID-19 pandemic [4,6]. This difference with other countries may be explained by the predominant provision of HIV care through specialized clinics that was deemed ‘essential care’ in the Netherlands, and was therefore not (or to limited extent) scaled down.

In addition, we found only moderate perceived shifts from face-to-face to online STI/HIV consultations. Internationally, this shift to telehealth applications seems higher [5,6]. However, Dutch private online providers, in general, saw an increase in online self-testing and STI/HIV service providers referred more often to them compared to pre-COVID. Private online providers indicated that they could have supported overburdened PSHS and GP services. However, a complicating factor was that professionals shared concerns about the quality of follow-up care after a positive result of private online remote testing that they would like to see improved.

Furthermore, we found that professionals were concerned to have lost sight of subpopulations in vulnerable circumstances. They, therefore, fear the pandemic may have magnified inequalities in access to STI/HIV care, which may be related to different levels of health and digital literacy required for telephone and video consultations. The perception of professionals that people in vulnerable circumstances may have been more likely to be missed is consistent with other international studies [14], which may be exacerbated by simultaneously discontinuing outreach activities, transitioning to online consultations and remote testing that in most cases are not covered by insurance [15].

This study has several limitations that need to be considered. The response was lower than aimed for, both for the survey and the interviews, despite our efforts to strengthen recruitment. Participation was especially limited for professionals in GP practices who indicated being overburdened by the end of the COVID-19 pandemic due to catch-up of missed care and capacity needed for care during the influenza season. Moreover, due to the heterogeneity of the interviewees’ professional roles, we are not sure if we have reached conceptual saturation. In addition, professionals who are interested in STI/HIV care may have been more likely to participate. The sample is hence not representative of all STI/HIV care providers in the Netherlands, and findings may not be generalizable to all STI/HIV care settings in the Netherlands or elsewhere. Also, the retrospective design may have resulted in recall bias, and interview findings could be affected by social desirability bias. Care was taken, however, to ensure the privacy and confidentiality of the interviews and surveys were anonymous.

To minimize inequalities in access to STI/HIV care during a pandemic or other public health crisis, it is recommended to (partially) reserve services for people in vulnerable circumstances, such as recent migrants and people with low health or digital literacy. Our study also suggests that efforts are needed to improve IT infrastructure to facilitate online and hybrid forms of care provision. Follow-up care after remote testing is an issue to be addressed, notably in guidelines for private providers. More collaboration between and within healthcare sectors proving STI/HIV care may help in better organizing this care and prevent overburdening of specific sectors. 

In conclusion, the study findings highlight how professionals experienced the impact of COVID-19 measures on the provision of STI/HIV care, access to care for vulnerable populations, and quality of care, adding detail and richness to surveillance data. The study findings in particular show how the COVID-19 pandemic differentially impacted different sectors of the Dutch STI/HIV care system and the specific challenges that healthcare professionals in these different sectors faced, and offers recommendations for STI/HIV care in potential future pandemics or other public health crises.

## Figures and Tables

**Table 1:** Mean scores and SDs for survey items for professionals in GP practices and PSHS for each of the four time periods.

	GP practices (n = 77)		PSHS (n = 93)		Total (n = 170)	
	M	SD	M	SD	M	SD
1. Access to care for priority populations						
In my practice/center, access to STI/HIV care for youth was… ^a^						
Period 1	2.16	0.96	1.29	0.69	1.68 ^2,3,4^	0.93
Period 2	2.71	0.71	2.01	0.76	2.33 ^1,4^	0.82
Period 3	2.64	0.62	1.96	0.85	2.26 ^1,4^	0.83
Period 4	2.95	0.56	2.66	0.94	2.79 ^1,2,3^	0.80
Total over periods	2.61	0.70	1.98	0.68	2.30 ***	0.65
In my practice/center, access to STI/HIV care for MSM was… ^a^						
Period 1	2.24	0.92	1.67	0.76	1.92 ^2,3,4^	0.88
Period 2	2.60	0.67	2.22	0.71	2.39 ^1,4^	0.71
Period 3	2.64	0.63	2.21	0.79	2.40 ^1,4^	0.75
Period 4	2.88	0.39	2.87	0.77	2.88 ^1,2,3^	0.63
Total over periods	2.59	0.70	2.24	0.67	2.42 **	0.78
In my practice/center, access to STI/HIV care for sex workers was… ^a^						
Period 1	2.55	0.81	1.76	0.80	2.02	0.88
Period 2	2.74	0.63	2.26	0.77	2.42	0.76
Period 3	2.81	0.48	2.15	0.77	2.37	0.75
Period 4	2.97	0.18	2.69	0.64	2.78	0.55
Total over periods	2.78	0.88	2.21	0.67	2.49 **	0.78
2. Demand and availability of STI/HIV care						
The demand for STI/HIV care was larger than we could offer ^b^						
Period 1	1.82	0.80	4.42	1.09	3.24	1.62
Period 2	1.88	0.80	4.20	1.00	3.15	1.48
Period 3	1.92	0.83	4.23	1.16	3.18	1.54
Period 4	2.02	0.96	3.88	1.20	3.04	1.43
Total over periods	1.91	1.08	4.18	1.09	3.05 ***	1.10
Less people received the STI/HIV care that they needed ^b^						
Period 1	2.87	1.31	4.24	1.17	3.63 ^2,4^	1.41
Period 2	2.70	1.20	3.98	1.03	3.41 ^1,4^	1.28
Period 3	2.72	1.21	3.95	1.18	3.40 ^4^	1.33
Period 4	2.60	1.14	3.50	1.22	3.10 ^1,2,3^	1.26
Total over periods	2.72	1.35	3.92	1.34	3.32 ***	1.36
3. Shifts to online counseling and care						
In my practice/center, the number of face-to-face consultations for STI/HIV care was… ^a^						
Period 1	1.38	0.71	1.26	0.75	1.32 ^2,3,4^	0.73
Period 2	2.43	0.90	2.03	0.90	2.21 ^1,3,4^	0.92
Period 3	2.15	0.83	1.81	0.83	1.96 ^1,2,4^	0.85
Period 4	3.28	0.84	2.63	1.04	2.92 ^1,2,3^	1.01
Total over periods	2.31	0.70	1.93	0.77	2.12 **	0.78
In my practice/center, the number of telephone or online consultations was… ^a^						
Period 1	3.56	1.66	3.64	1.48	3.60	1.57
Period 2	3.37	1.20	3.35	1.19	3.35	1.19
Period 3	3.49	1.25	3.51	1.22	3.50	1.23
Period 4	3.51	0.92	3.38	0.86	3.44	0.89
Total over periods	3.48	1.23	3.46	1.25	3.47	1.30
In my practice/center, referring to online STI/HIV testing by private providers was… ^a^						
Period 1	2.64	0.90	4.04	1.04	3.37	1.20
Period 2	2.68	0.66	3.76	0.84	3.24	0.93
Period 3	2.74	0.68	3.75	0.96	3.27	0.97
Period 4	2.89	0.60	3.27	0.97	3.09	0.71
Total over periods	2.74	0.79	3.71	0.87	3.22 ***	0.92
In my practice/center, the space for personal STI/HIV counseling was… ^a^						
Period 1	2.15	0.96	1.59	0.75	1.85 ^2,3,4^	0.90
Period 2	2.67	0.65	2.28	0.70	2.46 ^1,4^	0.70
Period 3	2.56	0.67	2.12	0.73	2.33 ^1,4^	0.73
Period 4	2.87	0.66	2.72	0.70	2.79 ^1,2,3^	0.68
Total over periods	2.56	0.68	2.18	0.70	2.37 ***	0.69
STI/HIV care has become more efficient ^a^						
Period 1	2.58	1.06	2.65	1.24	2.62 ^2,3,4^	1.16
Period 2	2.74	1.06	2.87	1.16	2.82 ^1,4^	1.11
Period 3	2.79	1.14	2.89	1.15	2.85 ^1,4^	1.14
Period 4	2.84	1.15	3.04	1.19	2.96 ^1^	1.17
Total over periods	2.74	1.09	2.86	1.09	2.80	1.11
4. Quality assurance of STI/HIV care						
Quality of STI/HIV care was lower than before COVID-19 ^b^						
Period 1	2.82	1.30	3.26	1.53	3.07 ^2,3,4^	1.44
Period 2	2.43	1.04	2.84	1.36	2.66 ^1,4^	1.19
Period 3	2.45	1.02	2.84	1.36	2.67 ^1,4^	1.23
Period 4	2.32	1.07	2.47	1.40	2.41 ^1,2,3^	1.27
Total over periods	2.51	1.15	2.86	1.16	2.68	1.17
STI/HIV care to vulnerable populations was negatively impacted ^b^						
Period 1	3.37	1.15	4.44	0.90	3.98 ^2,3,4^	1.14
Period 2	3.05	1.09	4.15	1.00	3.67 ^1,4^	1.17
Period 3	2.98	1.07	4.23	1.06	3.68 ^1,4^	1.23
Period 4	2.95	0.99	3.79	1.21	3.42 ^1,2,3^	1.19
Total over periods	3.09	0.97	4.16	0.97	3.62 ***	0.98
People in vulnerable circumstances at elevated STI/HIV risk went off-radar more ^b^						
Period 1	2.93	1.23	4.23	0.98	3.59 ^2,3,4^	1.24
Period 2	2.66	1.06	3.85	0.89	3.32 ^1,4^	1.13
Period 3	2.61	1.07	3.85	0.89	3.30 ^1,4^	1.22
Period 4	2.44	1.00	3.50	1.09	3.03 ^1,2,3^	1.17
Total over periods	2.66	0.97	3.83	0.97	3.24 ***	0.97
My job satisfaction was… ^a^						
Period 1	2.19	1.00	2.14	0.92	2.16 ^2,4^	0.95
Period 2	2.62	0.88	2.56	0.82	2.59 ^1,3^	0.85
Period 3	2.23	0.80	2.37	0.94	2.30 ^2,4^	0.87
Period 4	2.43	0.91	2.65	0.83	2.55 ^1,3^	0.87
Total over periods	2.37	0.83	2.43	0.88	2.40	0.86
In my practice/center, the opportunities for mentoring and professional education were… ^a^						
Period 1	2.02	1.02	1.56	0.88	1.77 ^2,3,4^	0.97
Period 2	2.38	0.94	1.86	0.73	2.09 ^1,4^	0.86
Period 3	2.35	0.89	1.92	0.79	2.11 ^1,4^	0.86
Period 4	2.69	0.72	2.37	0.67	2.51 ^1,2,3^	0.71
Total over periods	2.36	0.86	1.93	0.85	2.14 **	0.86
In my practice/center, the workload was… ^a^						
Period 1	2.97	1.49	3.72	1.46	3.34 ^3,4^	1.52
Period 2	3.71	0.94	3.54	1.20	3.63 ^3,4^	1.07
Period 3	4.17	0.94	3.60	1.18	3.89 ^1,2,4^	1.10
Period 4	4.43	0.78	3.95	1.04	4.19 ^1,2,3^	0.95
Total over periods	3.82	1.02	3.70	1.13	3.76	1.08

^a^ answering scale: 1 = much less, 2 = slightly less, 3 = equal, 4 = slightly more, 5 = much more—compared to before the pandemic. ^b^ answering scale: 1 = strongly disagree 2 = slightly disagree 3 = neutral 4 = slightly agree 5 = strongly agree—compared to before the pandemic. ** *p* < 0.01, *** *p* < 0.001 (between the two groups of professionals). ^1^ Significant difference (*p* < 0.05) with period 1, ^2^ significant difference with period 2, ^3^ significant difference with period 3, ^4^ significant difference with period 4 (all Bonferroni corrected).

**Table 2:** Quotes from interviews with STI/HIV providers per subtopic.

GP Practice	PSHS	HIV Clinic	Private Online Testing Provider
1. Access to STI/HIV care for priority populations			
“Those very vulnerable groups, the question is whether we really saw them. At one point I had someone at the consultation […]. That turned out to be someone who had had paid sex. It turned out she had been walking around with trichomonas all that time, but she didn’t dare to come because it was corona time. She had quite a lot of symptoms and yes, also a risk of passing it on of course. Yes, I found that quite shocking.”	“We [normally] do a lot of outreach, of course. So, we go […] to sex workers, to a group that stands up for lgbti+ interests, or to [agency X] for women who are here illegally. There are all kinds of projects for that, but they’re just temporarily shut down.”	“I notice that the PSHS is still busy with COVID, so my HIV-patients […] have nowhere to go. GPs often can’t, don’t know, do not take anal swabs, the PSHS has no space, and my first spot here at dermatology is end of November.” “What I said: refugees, there are a lot of MSM, and HIV problems are common. And they can’t find us. […] Actually, we had to learn that it was not the best decision, to close [outreach] immediately, when the corona crisis started.”	“Before corona we saw more low-risk, more young people and ‘security seekers’. […] In the corona period we saw more MSM. We saw that in the test results: there was more syphilis, more anal tests were ordered by men, which heterosexual men don’t do.” “Also the percentage of people that contacted [the client service] increased. Because these people were used to someone explaining the procedures. And now they had to do it themselves. So they just had more questions.”
2. Demand and availability of STI/HIV care			
“I think a large part of the men started to limit their contacts, and many did not have any contacts whatsoever. So on the one hand, the demand was lowered, but on the other hand, we started delivering more care to other people.” “People also rather didn’t want to go sit in the waiting room. […] What I hear back, is ‘actually I had these complaints a bit longer already’, like a contact bleeding, but they thought ‘maybe it will pass’.”	“Because of the lockdown, very few men were still having sex. Because everybody stayed home. In the first lockdown, we all had the motivation to stay home. And I really think that there was less sex.”	“It’s not the case that we received more people in the clinic saying they could not get PrEP.”	“My dream would be that you have a network with GPs and PSHS and the right online providers. That wherever you want to test, you get the right and good care. That they work together. That if there is a positive gonorrhea test in one part of the country, that you can go to your GP for treatment but can test with us.”
3. Shifts to online counseling and care			
“The module we use is not very user friendly. So it goes both ways. The patient had a hard time filling out the application, and the doctor had a hard time getting it operational.”	“We also had a lot of technical problems. The wifi did not work, the connection failed, and you think ‘man, what a hassle’. For the same amount of money I could just visit [the client].”	“I think that because of COVID, things have gained momentum. […] The measuring of your own blood pressure and weight at home, that is also increased. But to be honest, my impression is that this is mainly the case for the health empowered, higher educated, white gay men, to put it bluntly. There is a large group that does not appreciate this. […] In the end it is not about us, but what your client prefers most.”	“What I also think is that apart from the PSHS closing, people also had a fear and discomfort of bothering the GP […]. “I’ll get it tested myself” was said frequently, and there was more active referral. It’s more accepted [to get tested at a private provider].” “Actually, this [online testing] really took off because of COVID. […] A GP that did not want to do [a test] and asked ‘I want you to have complaints before you come here’. Well, you know, asymptomatic infections are common with STIs.”
4. Quality assurance of STI/HIV care			
“Well, if a GP practice has to organize vaccination in addition to actually catching up on care for people with complaints who have often waited too long, who need all kinds of complex care…”	“And then very occasionally, I have a man who has syphilis and is a sex worker, and he asks, “is PrEP something for me?”. Yes! And then he asks: ‘When can I start?’, ‘Yesterday’, I say. And then I [prescribe] PrEP, and then he goes again, and then he disappears again. Then he goes off the radar. Then he moves house, and then he doesn’t come anymore. So I struggle to get the vulnerable people to stay with us. The non-vulnerable people, they will come. I don’t worry about them.”	“You notice that, instead of half an hour face-to-face [consultation], in which you also take non-verbal attitude and mental wellbeing into account, we now telephoned for 5 minutes, in which you ask ‘how is it going’, talk about COVID, discuss the lab results for people with HIV, and then make an appointment for in six months, thinking, at the time, that it would be face-to-face.”	“Look at those commercial corona ‘test streets’ that now have limited demand of clients, they are now throwing themselves en masse into STI testing. We are genuinely concerned about that, because are they going to offer the quality needed, if you offer these kind of diagnostic tests? That’s almost impossible.”

## Data Availability

Survey data is available on request. Qualitative data is unavailable due to privacy or ethical restrictions.
